# Room temperature electrocompetent bacterial cells improve DNA transformation and recombineering efficiency

**DOI:** 10.1038/srep24648

**Published:** 2016-04-20

**Authors:** Qiang Tu, Jia Yin, Jun Fu, Jennifer Herrmann, Yuezhong Li, Yulong Yin, A. Francis Stewart, Rolf Müller, Youming Zhang

**Affiliations:** 1 Shandong University–Helmholtz Institute of Biotechnology, State Key Laboratory of Microbial Technology, School of Life Sciences, Shandong University, Shanda Nanlu 27, 250100 Jinan, People’s Republic of China; 2 Department of Microbial Natural Products, Helmholtz Institute for Pharmaceutical Research Saarland, Helmholtz Centre for Infection Research and Department of Pharmaceutical Biotechnology, Saarland University, Campus E8.1, 66123 Saarbrücken, Germany; 3 Department of Genomics, Dresden University of Technology, BioInnovations-Zentrum, Tatzberg 47-51, 01307 Dresden, Germany; 4 Animal Nutrition and Human Health Laboratory, College of Life Science of Hunan Normal University, 410081 Changsha, People’s Republic of China

## Abstract

Bacterial competent cells are essential for cloning, construction of DNA libraries, and mutagenesis in every molecular biology laboratory. Among various transformation methods, electroporation is found to own the best transformation efficiency. Previous electroporation methods are based on washing and electroporating the bacterial cells in ice-cold condition that make them fragile and prone to death. Here we present simple temperature shift based methods that improve DNA transformation and recombineering efficiency in *E. coli* and several other gram-negative bacteria thereby economizing time and cost. Increased transformation efficiency of large DNA molecules is a significant advantage that might facilitate the cloning of large fragments from genomic DNA preparations and metagenomics samples.

Usage of various competent cells in different molecular biology techniques such as cloning, amplification of plasmid DNA, construction of genomic libraries, gene expression, and mutagenesis are the routine procedures in each laboratory. Most commonly and extensively used bacterial strain is the Gram-negative bacterium *Escherichia coli* 1,2.

*E. coli* cells can be made competent by washing with divalent cations such as Ca^2+^ at 0 °C or under ice-cold conditions^3^,^4^. However, such metal ion liquids washed competent cells would have lower transformation efficiency than using the electroporation method. In the electroshock methods (electroporation transformation), high-voltage pulse treated *E. coli* cells become exceptionally competent after washing with ice-cold 10% glycerol or water^4^,^5^,^6^,^7^,^8^. The high voltage causes the cellular membrane to be transiently permeabilized, allowing the foreign material to enter into the cells^9^. High efficient electrocompetent cells are mainly used in library construction, mutagenesis and recombineering^10^. Protocols for electroporating Gram-negative bacteria including *E. coli* have already been described by many researchers^4^,^5^,^6^,^9^,^11^,^12^. Generally, cells are grown up to a suitable density, harvested, and followed by a series of washes to remove culture medium. Several factors have been identified to cause potential impact on the efficiency of electroporation transformation process. These factors include the electrical field strength, pulse decay time, pulse shape, temperature, type of cell, type of suspension buffer, concentration and size of the nucleic acid to be transferred^9^,^13^. According to the methods reported earlier, electrocompetent cell preparation have to be performed at ice-cold temperature and the equipment and washing solutions have to be maintained at the same temperature as well^14^,^15^,^16^.

Recombineering is now an alternative technology for conventional recombinant DNA engineering, a unique tool for large size DNA engineering, as well as the most appealing method of choice for bacterial genome engineering^17^,^18^. There are two main recombineering activities: one is based on linear plus circular homologous recombination (LCHR) initiated by the Red operon from λ phage^19^, and the other is the linear plus linear homologous recombination (LLHR) which is initiated by RecE/RecT from Rac phage^17^. LCHR is mainly applied to engineer plasmids which includes Bacterial Artificial Chromosome (BAC) while LLHR is primarily applied to linear DNA cloning (PCR cloning)^20^ and direct cloning^17^. Direct cloning is a shortcut for cloning of a large DNA fragments from genomic DNA without library construction and screening. For accomplishing the direct cloning, the DNA segment of interest should meet the linear cloning vector in one cell and then recombine each other. Therefore, the transformation efficiency and homologous recombination efficiency in the RecET proficient cells become the major limitation.

Keeping the cells cold was the pivotal point in the most of the protocols for electroporating Gram-negative bacterial strains including *E. coli* but there was no detailed explanations why this is important^16^. However, an improved transformation efficacy in the pathogen *Pseudomonas aeruginosa* when cells were washed at room temperature (RT) had previously been reported^21^. We surprisingly discovered that electrocompetent cells could be prepared at room temperature so that the cooling steps would be omitted. This was really astonishing because the conventional preparation method of electrocompetent cells for Gram-negative bacteria must be performed at 4 °C or preferably at 0–2 °C. Additionally, we found that the efficiency of direct cloning which was mediated by RecET recombineering would be dramatically improved by using the electrocompetent cells prepared at room temperature (named as room temperature competent cells). This astonishing discovery permitted the preparation and distribution of electrocompetent cells at a higher temperature. Here we present a novel DNA transformation method that is simplified, fast, efficient, convenient, and cost effective. This simple procedure does not only improves electroporation transformation efficiency in *E. coli* but also has implications for other bacterial hosts, e.g. *Agrobacterium*^22^, *Burkholderia*^13^, *Photorhabdus*^23^ and *Xenorhabdus*^23^.

## Results

### Effect of temperature shift on electrocompetent cells

**Figure 1:**
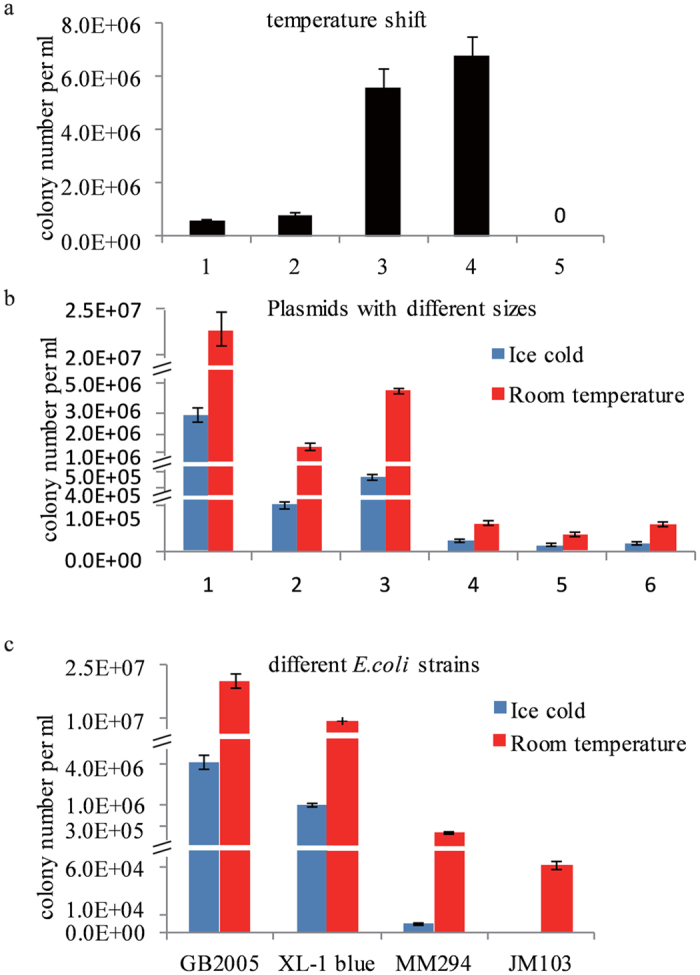
Transformation efficiency of competent cells. (**a**) Effect of temperature, *E. coli* GB2005 cells transformed by ~0.1 μg of pGB-amp-Ptet-plu1880 (27.8 kb) were plated on Amp plates. 1, the normal ice-cold method for preparing electrocompetent cells; 2, as for 1 but the cells were kept on ice for 15 min before electroporation; 3, as for 1 but the cells were placed at room temperature (RT) for 15min before electroporation; all cuvettes were used at RT; 4, every step was done at RT; 5, no plasmid DNA. (**b**) RT prepared cells were transformed with different plasmids. 1, pBR322 origin with ampicillin resistance (27.8 kb); 2, p15A origin with chloramphenicol resistant (54.7 kb); 3, p15A origin with ampicillin resistance (54.7 kb); 4, BAC with chloramphenicol resistant (>120 kb); 5, BAC with kanamycin resistant (91.7 kb); 6, BAC with ampicillin resistant (91.7 kb). (**c**) Different *E. coli* strains tested for electroporation transformation. Cells were transformed by 0.1 μg of pGB-amp-Ptet-plu1880 and plated on Amp plates. Error bars, SD; n = 3.

It was inconvenient to maintain low temperature conditions for preparation, storage and transport of the electrocompetent cells. We intended to test the transformation efficiency of the electrocompetent cells prepared at room temperature. A large plasmid pGB-amp-Ptet-plu1880 (27.8 kb) was transformed into *E. coli* GB2005 strain^17^,^24^ at various temperature. The warm electrocompetent cells showed 10 times higher transformation efficiency than the cold electrocompetent cells (Fig. 1a). After placing the cold electrocompetent cells at room temperature for 15 minutes, the transformation efficiency increased by 5 folds (Fig. S1a). In contrast, after the room temperature electrocompetent cells were placed on ice for 15 minutes before electroporation, there was a significant decrease in transformation efficiency (Fig. S1b).

The room temperature in our laboratory was set at 24 °C. To determine the range of optimum temperature for the preparation of competent cells, we prepared the cells at different temperature ranges and revealed that the best temperature for electrocompetent cell preparation was in the range of 24°–28 °C (Fig. S2).

### Effect of different plasmids on electrocompetent cells

Plasmids were varied in the size, selection marker and origins of replication. Initially we tested three plasmids with different sizes. Two of them were p15A origin plasmids with ampicillin (amp) or chloramphenicol (cm) resistance. Another one was a pBR322 origin based plasmid with ampicillin resistance. All the plasmids gained higher transformation efficiency with room temperature electrocompetent cells (Fig. 1b, column 1–3). We also tested BAC vectors with different size and selection markers. All BACs gained higher transformation efficiency when room temperature electrocompetent *E. coli* GB2005 cells were used (Fig. 1b, column 4–6). These results indicated that for electrocompetent transformation, room temperature electrocompetent cells were more efficient than cold electrocompetent cells irrespective of their size, selection marker and origins of replication. Therefore the room temperature electrocompetent cells could be a better candidate for gene cloning, construction of DNA libraries and mutagenesis than cold electrocompetent ones.

### Effect of different strains on electrocompetent cells

The *E. coli* GB2005 was an optimized strain for plasmid transformation and propagation^17^,^25^. Along with this strain, several other commonly used *E. coli* strains were also tested for room temperature transformation as well. Results revealed that although different *E. coli* strains varied in their relative transformation efficiencies, all of them exhibited higher transformation efficiency when their electrocompetent cells were prepared at room temperature (Fig. 1c). We also tested the improving approach in a few of other Gram-negative bacterial strains. *Burkholderia glumae* PG1 was an industrial strain for detergent lipidase production^26^, which could also be the heterologous host used for PKS/NRPS gene clusters expression (unpublished data). An oriV origin plasmid pRK_2_-apra-kan based on plasmid pBC301^27^,^28^, was utilized for transformation. When PG1 competent cells were prepared at room temperature, the electroporation efficiency of RK_2_ plasmid was around three times higher than the cells prepared on ice (Fig. S3a). Other Gram-negative bacterial strains, such as *Agrobacterium*^22^*, Burkholderia*^13^*, Photorhabdus*^23^ and *Xenorhabdus*^23^, were tested for RK_2_ plasmid transformation by using room temperature and cold temperature protocols. All the results indicated that room temperature competent cells had higher transformation efficiency than cold competent cells (Fig. S3b).

### Improvement of recombineering by using room temperature electrocompetent cells

**Figure 2:**
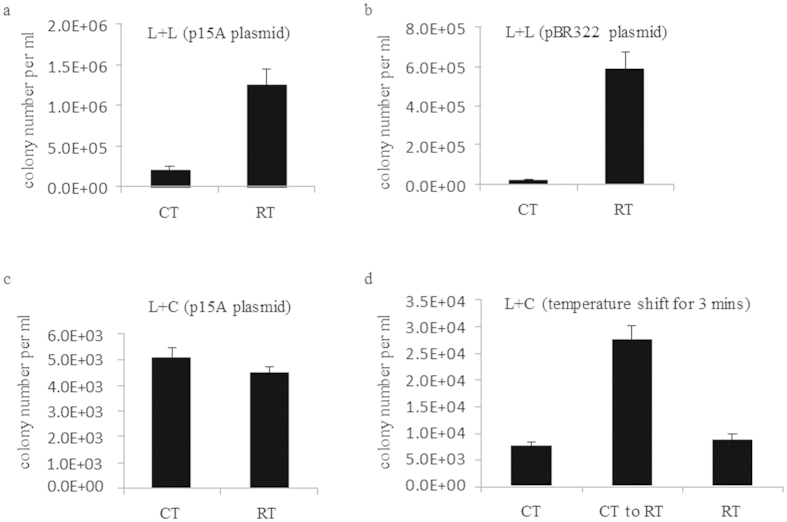
Recombineering using room temperature electrocompetent cells. (**a**) Colony number of a standard LLHR assay^17^ in GB05-dir from the normal and the cold method in *E. coli*. (**b**) As for A, but with pBR322 origin. (**c**) As for A, but with a standard LCHR assay in GB05-red. (**d**) The electrocompetent cells were prepared on ice first. After adding PCR product kan cassette into the ice-cold electrocompetent cells, the cells plus DNA mixture were shifted to RT for 3 minutes before electroporation (middle column). CT, Cold temperature; RT, Room temperature. Error bars, SD; n = 3.

The plasmid transformation efficiency significantly increased by room temperature electrocompetent cells was not the destination. It was necessary to evaluate the improvement of the room temperature protocol on lambda Red or Rac RecET mediated recombineering. A simple assay using a PCR product of linear vector (p15A ori plus cm or pBR322 ori plus cm) and a PCR product with kanamycin (kan) was built to test LLHR efficiency^17^. *E. coli* strain GB05-dir with *rec*ET on its chromosome was used for LLHR test^25^. The results showed that LLHR in room temperature competent cells was 6–10 times more efficient than the cells prepared on ice. Both p15A origin and pBR322 origin plasmids gained the same fold increase (Fig. 2a,b). A direct cloning experiment to fish out the thailandepsin gene cluster (~39 kb) from *Burkholderia thailandensis* had been performed, around 150 colonies were obtained by using cold electrocompetent cells, but by using room temperature electrocompetent cells more than 600 colonies were obtained. This improvement leads to a higher chance to clone large DNA fragments from genomic DNA pools directly.

PCR cloning is a routine exercise in every molecular biology laboratory^20^. It is thus our interest to find out an easy and inexpensive way to clone PCR products. Since the electrocompetent cells prepared at room temperature improves the LLHR efficiency around 10 folds, it is essential to find out the minimum homology sequences needed for LLHR. Previously, we identified 20 bp as the minimum length of sequence homology required for recombineering^29^,^30^. To test whether the minimal length could be further shortened, pBAD24 vector was digested with EcoR I/Hind III as linear recipient, and PCR product cassette (Tn5-neo) flanked with short homology arms to the ends of digested pBAD24 vector was used as linear donor fragment (Fig. S4). Seven PCR products with different sizes of homology arms (HA) were designed to test the LLHR efficiency. Results revealed that only 8 bp of terminal homology was sufficient via room temperature protocol (Fig. S5). When ice-cold cells were used, the minimum homology arms required for recombineering were found 12 bp. These data indicated that LLHR might be used to generate a kit for PCR product or small DNA fragment cloning by using homology arms as short as 8 bp.

In contrast to the LLHR experiment, LCHR efficiency was not increasing in the room temperature protocol when compared to the cold protocol (Fig. 2c). However, we discovered that LCHR efficiency would be significantly raised when freshly prepared cold electrocompetent cells were placed at room temperature for 3 minutes (Fig. 2d), suggesting that transient swelling of the cells had a beneficial effect.

### Stability of room temperature electrocompetent cells

Normally, after 2.5–3.0 hours cultivation at 37 °C, *E. coli* GB2005 reached OD_600_ 0.4–0.6 which was in the log phase, the period with the best transformation efficiency of the cells. When bacterial cells were overgrown, the transformation efficiency dropped down (Fig. S6a), and the transformation efficiency of the cold electrocompetent cells was completely lost after 4 hours or 6 hours (only 18 and 5 colonies respectively) (Fig. S6a). But room temperature electrocompetent cells still kept relatively high efficiency even after 4 or 6 hours cultivation. *E. coli* GB2005 cultured for 4 hours at 37 °C reached OD_600_ 1.0 ~ 1.2 and cultured for 6 hours reached OD_600_ > 1.8 which was at the plateau phase. It was noteworthy that over-grown or even overnight cultured bacterial cells could still be used for transformation when room temperature protocol was used for preparing competent cells.

To predigest the transformation process, we had tested whether the recovery step could be omitted. For simple plasmid transformation, the recovery step could be omitted when the electrocompetent cells were prepared by using room temperature protocol (Fig. S6b). Although the transformation efficiency in the un-recovery room temperature group was around 30% less than in the recovery room temperature group, it was still at least 5 times higher comparing to the cold temperature group, either recovery or not. Results suggested that plasmid or ligation transformation could be performed in a few minutes after electroporation by using room temperature competent cells. Previous results concluded that the room temperature electrocompetent cells had much better transformation efficiency than cold electrocompetent cells. Furthermore we wanted to know how long the competent cells could stay at room temperature without any significant loss of transformation efficiency. Results showed that room temperature competent cells lost around 30% efficiency after 1 hour storage at room temperature, around 60% lost after 4 hours and around 80% lost after one day (Fig. S7). These results indicated that the room temperature competent cells lost their transformation efficiency to the maximum when stored in room temperature more than one day. To avoid this efficiency loss, room temperature competent cells were prepared by using 10% glycerol^11^ and dried by vacuum and stored at 4 °C till three days. Result showed that dried room temperature competent cells prepared in 10% glycerol lost their 55% efficiency as compared to the room temperature competent cells without dry (Table S2). But interestingly, the dried competent cells prepared in 10% glycerol could keep the LLHR efficiency up to 3 days without any further efficiency loss (Table S3). This ability gives us an opportunity in the future to deliver the competent cells in routine cooling pack, which is easier and cost effective.

### Electron microscopy analysis of competent cells

**Figure 3:**
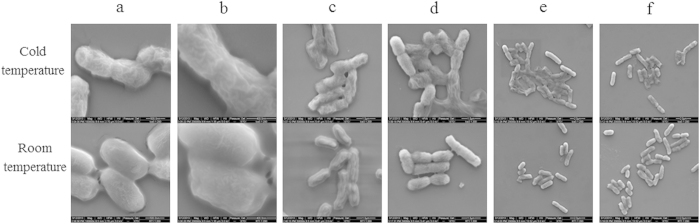
Phenotypes of the cells by room temperature and cold protocols and also electroporated the cells for subsequent analysis by electron microscopy. (**a**–**d**): micrograph of the cells washed with cold and room temperature protocols with different magnications. (**e**–**f**): micrograph of the electroporated cells mixture with episomal insertion DNA.

To find the reasons of higher efficiency in room temperature protocol, electron microscopy was used for comparative analysis of the morphological shapes of cold competent cells and room temperature competent cells of *E. coli*. Their comparative analysis showed that cold competent cells appeared to shrink more than room temperature cells, and the surface of room temperature competent cells was found smoother (Fig 3a–d). Shrunken cells might be more difficult to transform, and the bacterial cell membrane and wall could be more permeable for foreign DNA entry at a higher temperature. Additionally, it may be difficult for the shrunken cells to form pores that allow DNA transfer through the cell membrane under electroporation conditions, and after electroporation most of the cold competent cells were found to be lysed. (Fig. 3e,f). From this we assume that the bacterial cell membrane/cell wall might have better permeability for foreign material to enter into the cell.

## Discussion

The ability to introduce exogenous DNA molecules into the cells plays key role in the development of molecular biology techniques, such as mutagenesis and genetic engineering of microorganisms. Several methods have been reported to introduce exogenous DNA molecules into the cells which includes chemical treatment, electroporation, utilization of a biolistic gun, polyethylene glycol, ultrasound, microwave, and hydrogel^31^. In those methods, electroporation has been often demonstrated to be more efficient and convenient way to transform a large number of microorganisms used for genetic studies^32^, and many efforts have been performed to increase its efficiency^33^,^34^,^35^,^36^.

The phage-derived homologous recombination systems have been developed into very useful DNA engineering technologies, well known as recombineering which has also been performed in electrocompetent cells^10^,^20^. This suggests that a crucial step in recombineering is the transformation of *E. coli* by electroporation.

In conventional electroporation transformation, the electrocompetent cells were prepared on ice and the other supplies were also in cold environment, including pre-chilled cuvette, buffer, and centrifuge. The cells must be repeatedly washed before electroporation to remove conductive solutes. If the conductivity of a cell mixture is too high, then arcing will occur during electroporation, which will ruin the experiment. The washing process can elicit a stress response that can lead to decrease in transformation efficiency. If the cells are kept at 4 °C then they are inactive and this stress response is prevented. However, current studies reveals that the electroporation transformation efficiency is decreased at ice-cold temperature (Fig. 1). This decreased efficiency might be due to ice cold temperature which alters the cell membrane topology. The cell membrane mainly consists of phospholipids and proteins^37^ and the phospholipid bilayer forms a stable barrier between two aqueous compartments. Embedded proteins of phospholipid bilayer carry out the specific functions of the plasma membrane, including the selective transportation of molecules across the membrane and cell-cell recognition^38^. At ice-cold temperature, the fatty acid tails of the phospholipids become more rigid. This affects the fluidity, permeability, and the cell’s ability to live^39^. Therefore, cold temperatures may be not favourable for the survival and thus decreases the transformation efficiency. Additionally, during electroporation process due to externally applied electric field there is a significant increase in the permeability of cell’s plasma membrane , which is used to introduce exogenous DNA into bacterial cell^32^. Previous reports revealed that if the environmental conditions were changed, including the temperature, the cell membranes undergoes a gross morphological changes^40^. These structural perturbations were associated with characteristic disturbances of functions such as loss of selective permeability. Similar results were observed in this study that cold competent cells were appeared to shrink more than room temperature cells, and additionally more cold competent cells were found lysed after electroporation (Fig. 3).

The temperature effects on electroporation transformation could be explained by thermal effects during electro pore formation^41^,^42^,^43^. According to the electroporation theory, hydrophobic pores in the cell membrane were formed spontaneously by lateral thermal fluctuations of the lipid molecules^39^, which suggested that hydrophobic pores formation would be enhanced by increased temperature conditions. To improve recombination efficiency many parameters had been described previously^17^,^44^,^45^ except the transformation efficiency. This study showed that LLHR efficiency in room temperature competent cells was higher than in the same cells prepared on ice (Fig. 2a,b), but the room temperature protocol did not increase LCHR efficiency when compared to the cold protocol (Fig. 2c). The Red recombinases (Red alpha and beta) might be not stable while preparing the competent cells at room temperature.

In conclusion, this study reports an unexpected finding, that is contrary to common assumption, that it is better to prepare bacterial cells at room temperature than on ice for electroporation. In addition, this study also shows that this is not only efficient for *E. coli* but also for several other gram-negative and gram-positive hosts. However, further research will be essential to confirm the transfer and principle of membrane in competent cells.

## Methods

### Strains, plasmids and reagents

The bacterial strains and plasmids used in this study were listed in Table S1. The antibiotics were purchased from Invitrogen. *E. coli*, *Agrobacterium*, *Photorhabdus* and *Xenorhabdus* were cultured in Luria–Bertani (LB) broth or on LB agar plates (1.2% agar) with ampicillin [*amp*] (100 μg/mL), kanamycin [*kan*] (15 μg/mL) or chloramphenicol [*cm*] (15 μg/mL) as required. *Burkholderia glumae* PG1 was cultured in MME medium (5 g/L K_2_HPO_4_, 1.75 g/L Na(NH_4_)HPO_4_ × 4H_2_O, 1 g/L Citrate, 0.1 g/L MgSO_4_ × 7 H_2_O, 8 g/L Glucose, pH7.0). *Burkholderia* DSM7029 was cultured in CYCG medium (6g/L Casitone, 1.4 g/L CaCl_2_ × 2 H_2_O, 2 g/L Yeast extract and 20 ml/L Glycerol).

### Preparation of electrocompetent cell at cold and room temperature conditions

The electrocompetent cells at cold temperature were prepared according to the protocol established previously in our lab^46^. For electrocompetent cells at room temperature, overnight culture were diluted into 1.4 mL LB medium and again cultured at 37 °C at 900 rpm in an Eppendorf ThermoMixer. After 2 hours of incubation when OD_600_ was approximately reached up to 0.6, the bacterial cells were centrifuged at 9000 rpm at room temperature (24 °C). The supernatant was then discarded and the cells were resuspended in 1 mL of dH_2_O at room temperature, and washing step was repeated. The bacterial cells were again resuspended in about 30 μl of dH_2_O (24 °C) and the tubes were placed at room temperature. 300 ng of the each plasmid DNA or PCR products were added into the prepared cells. The DNA-cell mixture were then transferred into 1 mm-gap cuvette (24 °C) for electroporation at 1250 volts. The cuvette was then flushed with 1 ml fresh medium and the cells were recovered by the incubation at 37 °C for 1 hour. In the end, the culture was streaked on the LB plates containing appropriate antibiotics.

### Preparation of electrocompetent cell to test the effect of different temperature range

*E. coli* GB2005 strain was cultured at 37 °C till OD_600_ was reached at 0.6. The cells were pelleted and washed by dH_2_O at different temperature range (2, 15, 20, 22, 24, 26, 28, 30, 32, 34 and 37 °C). The cuvettes were also kept at these temperatures. After electroporation with pGB-amp-Ptet-plu1880 plasmid, 1 ml LB was added into the cuvette to recover the transformed cells and then incubated at 37 °C. After 1 hour incubation 0.004 μl of cells (diluted by fresh LB) were streaked on LB plates containing ampicillin (100 μg/mL). The colonies were counted after 24 hours of cultivation.

### Recombineering assays

In LCHR assay we used a 2 kb p15A-cm plasmid carrying the chloramphenicol resistance gene and a 2 kb kan-PCR product carrying the kanamycin resistance gene. Each end of the kan-PCR product had a 50-bp homology arm to the p15A-cm plasmid between the chloramphenicol gene (cm) and the p15A origin. The circular plasmid (200 ng) and the PCR product (200 ng) were co-electroporated into *E. coli* GB2005 expressing the lambda Red recombinase to generate the chloramphenicol plus kanamycin-resistant plasmid p15A-cm-kan (4 kb). The expression plasmid was pSC101-BAD-gbaA-tet. The recombinants were selected on LB plates with double antibiotic selection.

One of LLHR assays was just like above mentioned setup except the p15A-cm plasmid was linearized between the two 50-bp homology arms. The linear plasmid backbone (200 ng) and the kan-PCR product (200 ng) were co-electroporated into *E. coli* GB2005 expressing the *rec*ET recombinase to generate the plasmid p15A-cm-kan. Another LLHR assay used EcoR I and Hind III digested 4.5 kb pBAD24 plasmid (450 ng) and 1,7 kb Tn5-neo PCR (170 ng) to generate the ampicillin plus kanamycin-resistant plasmid pBAD24-neo (6.2 kb). The expression plasmid was pSC101-BAD-ETgA-tet. The recombinants were selected on LB plates with double antibiotic selection.

Both kan-PCR and Tn5-neo-PCR were amplified from suicide R6K plasmid to avoid the selection background from the carryover of the PCR template (any residual circular plasmid). The negative control with DNA electroporation into uninduced cells was done to indicate the sufficient selection pressure. After colony counting, 8 clones from each of the triplicate experiments were picked up for plasmid DNA preparation and restriction analysis to prove the successful accomplishment of the recombineering experiment.

### Preparation of dried room temperature electrocompetent cell

The electrocompetent cells were washed twice with dH_2_O or 10% glycerol at room temperature, cells were pelleted once again and remaining dH_2_O or 10% glycerol was removed by pipetting. Cell pellet was dried under the vacuum for 30 min and stored at 4 °C. For transformation, dried cells were resuspended in 25 μL dH_2_O (without glycerol) at room temperature and DNA was added into the cells. Cells and DNA were electroporated at 1250 v by using 1 mm-gap electroporation cuvette and Eppendorf electroporator as usual.

### Cells preparations for Electron Microscope

*E. coli cells* were harvested by centrifugation and were fixed in 2% paraformaldehyde/1% glutaraldehyde for 20 min at room temperature. After repeated washing with ultrapure water the cell pellet was resuspended and a small aliquot of the samples in water was placed on a silicium waver and was dried under ambient conditions. Next, the *E. coli* cells were investigated with secondary electrons under high-vacuum conditions in an ESEM type FEI Quanta400 FEG at 5 kV accelerating voltage.

## Additional Information

**How to cite this article**: Tu, Q. *et al.* Room temperature electrocompetent bacterial cells improve DNA transformation and recombineering efficiency. *Sci. Rep.* **6**, 24648; doi: 10.1038/srep24648 (2016).

## Supplementary Material

Supplementary Information
