# Supplementary material for: Room temperature electrocompetent bacterial cells improve DNA transformation and recombineering efficiency
Source: Sci Rep. 2016 Apr 20;6:24648. doi: 10.1038/srep24648 (PMC4837392; doi:10.1038/srep24648)
Supplement: Supplementary Information [file srep24648-s1.doc]

**Supplemental Information**

**Room temperature electrocompetent bacterial cells improve DNA transformation and recombineering efficiency**

Qiang Tu, Jia Yin, Jun Fu, Jennifer Herrmann, Yuezhong Li, Yulong Yin, A. Francis Stewart, Rolf Müller and Youming Zhang

**Inventory of Supplemental Information**

**Supplemental data**

**Figure** **S1.** Temperature shift effect on the competent cells for transformation. On Page 5.

**Figure S2.** Effect of different temperature on electrocompetent cells. On Page 5.

**Figure S3.** Transformation efficiency comparison of warm and cold temperature in different gram-negative strains. On Page 6.

**Figure S4.** Diagram of LLHR by using short homology arms**.** On Page 7.

**Figure S5.** Effect of the length of homology arms on room temperature electrocompetent cells (warm cells). On Page 7.

**Figure S6.** Effect of over-grown cells on transformation efficiency and electroporation without recovery step. On Page 8.

**Figure S7.** Stability of room temperature electrocompetent cells stored at room temperature. On Page 8.

**Table S1** Strains and plasmids.

**Table S2** Transformation efficiency (colonies on plates with ampicillin (x10^4^)) using cells prepared in dH_2_O or 10% glycerol. On Page 9.

**Table S3** LLHR efficiency (colonies on plates with ampicillin and kanamycin) using cells prepared in dH_2_O or 10% glycerol. On Page 9.

**Supplemental references**


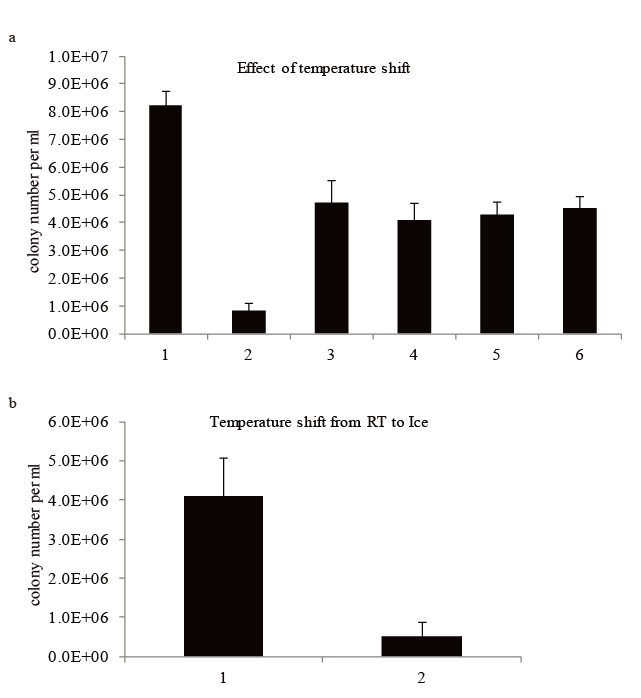


**Figure S1. Temperature shift effect on the competent cells for transformation.** (a) GB2005 cells transformed by ~0.1µg of pGB-Ptet-plu1880 (27.8kb) and plated on Amp plates. 1 -cells prepared at RT; 2 -cells prepared on ice; 3 -cells prepared on ice first then left at RT for 2.5min before electroporation; 4 -same as 3 but at RT for 4min; 5 -same as 4 but at RT for 10min; 6 -same as 3 but at RT for 15min. (b) 1 -cells prepared at RT; 2 -cells prepared at RT, then placed on ice for 15min before electroporation. Error bars, SD; n = 3.


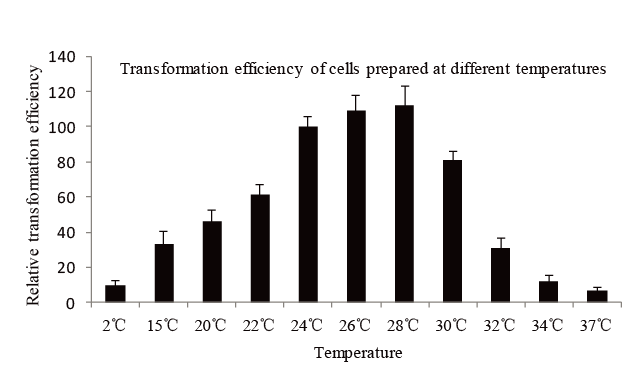


**Figure S2. Effect of different temperature on electrocompetent cells.** GB2005 cells were transformed by ~0.1µg of pGB-Ptet-plu1880 (27.8kb) and plated on Amp plates. Ice to 37^o^C were used for preparing competent cells and electroporation. It shows the results in relative transformation efficiency using the transformants at 24°C (room temperature) as standard (100%). Transformants from different temperatures were divided by standard to give the relative transformation efficiency. It also shows that significant results were obtained by preparing competent cells between 24°C-28°C. This confirms that preparation of electrocompetent cells can be made as simple as possible. Error bars, SD; n = 3.


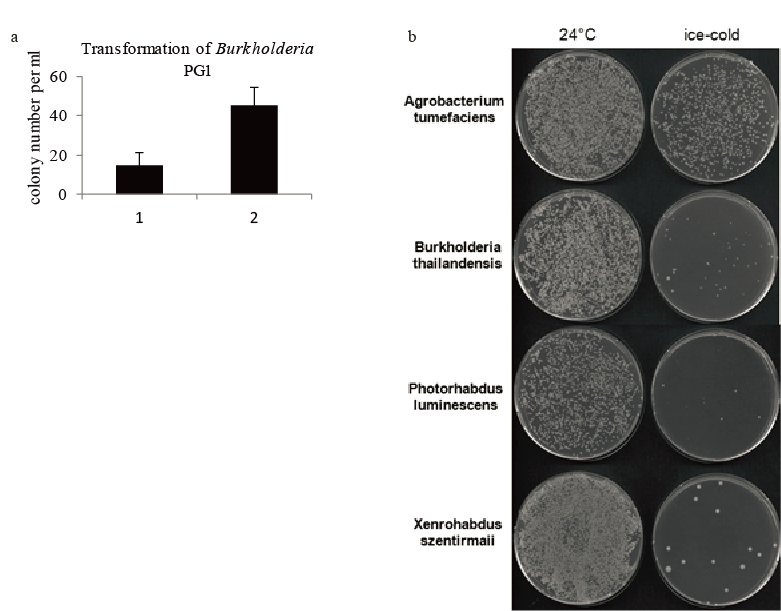


**Figure S3. Transformation efficiency comparison of warm and cold temperature in different** **gram-****negative strains. (a)** pRK2-apra-km plasmid was used to transform into *Burkholderia* PG1. The transformants were Km resistant. **(b)** A few bacterial strains: *Agrobacterium* (G^-^), *Burkholderia* DSM7029 (G^-^), *Photorhabdus* (G^-^), and *Xenorhabdus* (G^-^) were used to perform the transformation experiment. Error bars, SD; n = 3.


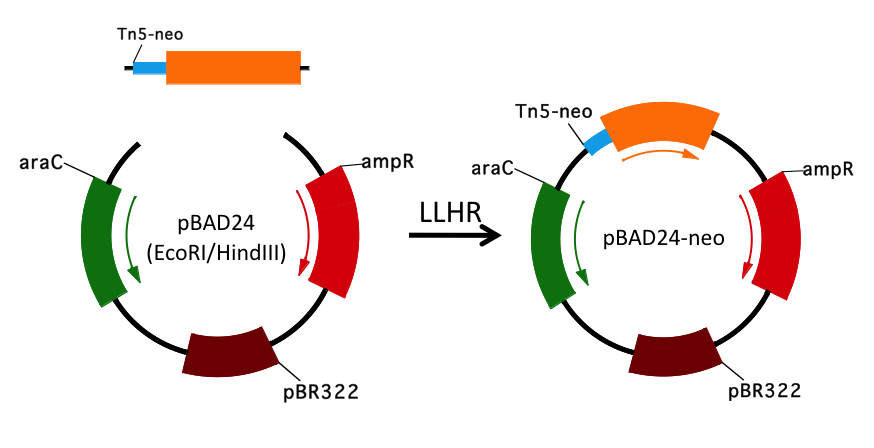


**Figure S4. Diagram of LLHR by using short homology arms.** pBAD24 circle vector digested by EcoR I plus Hind III was used as linear recipient vector. The homology sequences are exactly exposed at the ends. Tn5-neo PCR product flanked with short homology arms to the ends of digested pBAD24 vector is used as linear donor fragment.


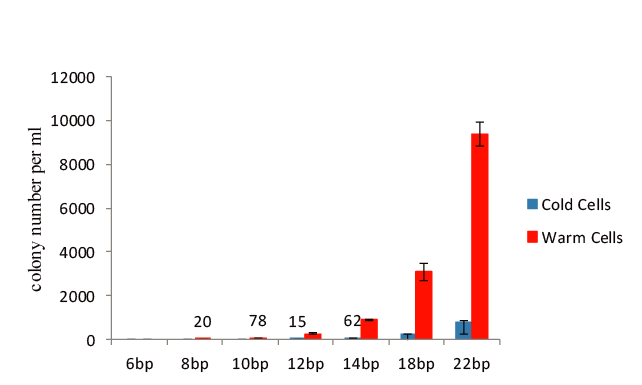


**Figure S5. Effect of the length of homology arms on room temperature electrocompetent cells (warm cells).** Seven PCR products with different homology arms (HA) were used for testing the LLHR efficiency. The homology arms can be as short as 8bp for LLHR to occur when cells were prepared at RT. When ice-cold cells used, the minimum homology arms are 12bp. Error bars, SD; n = 3.

**
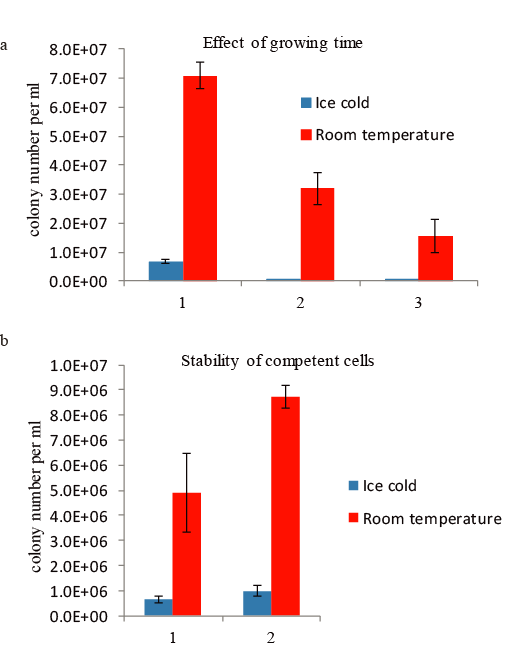
**

**Figure S6. Effect of over-grown cells on transformation efficiency and electroporation without recovery step.** **(a)** 35µL overnight cultured GB2005 cells were diluted into 1.4mL LB medium and cultured at 37°C for different time courses. Electrocompetent cells were transformed by 0.1µg of pGB-Ptet-plu1880 and plated on LB plates plus amp. 1 -cells growing for 2.5 hours, OD600=0.4; 2 -cells growing for 4 hours, OD600=1.2; 3 -cells growing for 6 hours, OD600=1.8. (**b**) GB2005 cells transformed by 0.1µg of pGB-Ptet-plu1880 and plated on Amp plates. 1 -cells plated directly after electroporation; 2 - same as 1 but after 1 hour recovery at 37^o^C. Error bars, SD; n = 3.


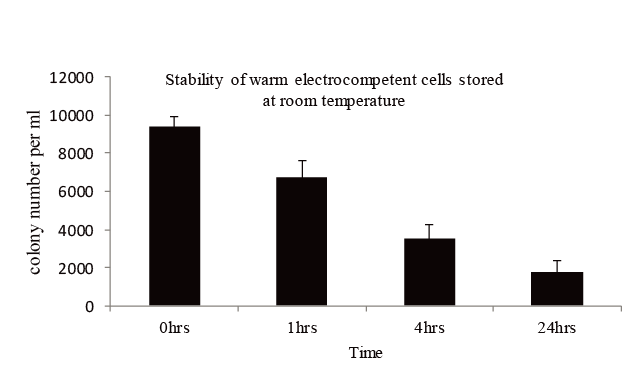


**Figure S7. Stability of room temperature electrocompetent cells stored at room temperature.** The room temperature competent cells lost around 30% of efficiency after 1 hour of storage at room temperature, 60% after 4 hours and approximately 80% after 1 day. Error bars, SD; n = 3.

**Table S1** Strains and plasmids.

| Strain or plasmid | Characteristics | References or sources |
| --- | --- | --- |
| Strains |  |  |
| *E. coli* GB05 | F-*mcr*A ∆(*mrr*-*hsd*RMS-*mcr*BC) *φ*80*lac*Z∆M15 ∆*lac*X74 *rec*A1 *end*A1 *ara*D139 ∆(*ara, leu*)7697 *gal*U *gal*K λ *rpsLnup*G*fhu*A::IS2 *rec*ET *redα*, phage T1-resistent | 1 |
| *E. coli* GB05-dir | GB2005, *ara*C-BAD-ETγA | 2 |
| *E. coli* GB05-red | GB2005, *ara*C-BAD-γβαA | 1 |
| *Burkholderia glumae* PG1 | lipidase -producing wild-type strain, host for heterologous expression of PKS/NRPS gene clusters | 3 |
| *Agrobacterium tumenfaciens* | gram-positive strain | 4 |
| *Burkholderia* DSM7029 | gram-negative strain | 3 |
| *Photorhabdus luminescens* | gram-negative strain | 5 |
| *Xenorhabdus stockiae* | gram-negative strain | 5 |
| Plasmid |  |  |
| pGB-amp-Ptet-plu1880 | pBR322 replicon, ampR | 6 |
| pRK2-apra-km | oriV origin, kmR | This study |
| pBC301 | oriV origin | 7,8 |
| pBeloBAC11-dis | BAC, kmR | This study |
| p15A-cm | p15A replicon, cmR | 2 |
| p15A-cm-km | p15A replicon, cmR, kmR | 2 |
| pBAD24 | pBR322 replicon, ampR | 9 |
| pBAD24-neo | pBR322 replicon, ampR, kmR | This study |

**Table S2** Transformation efficiency (colonies on plates with ampicillin (x10^4^)) using cells prepared in dH_2_O or 10% glycerol.

|  | Cells before dry | Dried cells day 0 | Dried cells day 1 | Dried cells day 3 |
| --- | --- | --- | --- | --- |
| dH_2_O | 640 | 0 | 0 | 0 |
| 10% glycerol | 468 | 196 | 212 | 188 |

**Table S3** LLHR efficiency (colonies on plates with ampicillin and kanamycin) using cells prepared in dH_2_O or 10% glycerol.

|  | Cells before dry | Dried cells day 0 | Dried cells day 1 | Dried cells day 3 |
| --- | --- | --- | --- | --- |
| dH_2_O | 420 | 0 | 0 | 0 |
| 10% glycerol | 360 | 298 | 272 | 284 |

References

1 Fu, J., Teucher, M., Anastassiadis, K., Skarnes, W. & Stewart, A. F. in Methods in Enzymology Vol. 477 (eds M. Wassarman Paul & M. Soriano Philippe) 125-144 (Academic Press, 2010).

2 Fu, J. *et al.* Full-length RecE enhances linear-linear homologous recombination and facilitates direct cloning for bioprospecting. *Nat. Biotechnol.* **30**, 440-446 (2012).

3 M, O. *et al.* Glidobactins A, B and C, new antitumor antibiotics. II. Structure elucidation. *J. Antibiot.* **41**, 1338–1350 (1988).

4 Hu, S. *et al.* Genome engineering of *Agrobacterium tumefaciens* using the lambda Red recombination system. *Appl. Environ. Microbiol.* **98**, 2165-2172 (2014).

5 Yin, J. *et al.* A new recombineering system for *Photorhabdus* and *Xenorhabdus*. *Nucleic Acids Res*. **43** (6), e36 (2015).

6 Bian, X. *et al.* Direct cloning, genetic engineering, and heterologous expression of the syringolin biosynthetic gene cluster in *E. coli* through Red/ET recombineering. *ChemBioChem* **13**, 1946-1952 (2012).

7 Xiang. A mini binary vector series for plant transformation. *Plant Mol. Biol.* **40**, 711-717 (1999).

8 Yang. Functional modulation of the geminivirus AL_2_ transcription factor and silencing suppressor by self-interaction. *J. Virol.* **81**, 11972–11981 (2007).

9 [Guzman, L.M](http://www.ncbi.nlm.nih.gov/pubmed/?term=Guzman LM%5BAuthor%5D&cauthor=true&cauthor_uid=7608087)., *et al*. Tight regulation, modulation, and high-level expression by vectors containing the arabinose pBAD promoter. [*J. Bacteriol*.](http://www.ncbi.nlm.nih.gov/pubmed?cmd=Search&term=7608087) **177**, 4121-4130 (1995).
